# Prehabilitation beyond the trimodal standard: occupational therapy in a quadrimodal cancer care model

**DOI:** 10.1007/s00520-026-10364-z

**Published:** 2026-02-26

**Authors:** Tomáš Brtnický, Markéta Polková, Marie Tichá, Peter Koliba, Oľga Dubová, Michal Zikán, Alena Jarolímková, Petr Hubka, Pavel Kabele, Jana Matějíčková, Petra Ovesná, Petra Sládková

**Affiliations:** 1 Department of Gynaecology and Obstetrics, Charles University - 1st Faculty of Medicine and Bulovka University Hospital, Prague, Czech Republic; 2 Department of Rehabilitation, Faculty of Biomedical Engineering, Bulovka University Hospital, Czech Technical University in Prague, Prague, Czech Republic; 3 Department of Clinical Psychology, Bulovka University Hospital, Prague, Czech Republic; 4 Institute of Biostatistics and Analyses, Faculty of Medicine, Masaryk University, Brno, Czech Republic; 5 Bulovka University Hospital, Budínova 67/2, Prague 8, Prague, 180 00, Czech Republic

**Keywords:** Prehabilitation, Frailty, Cancer surgery, Occupational therapy, Physiotherapy, Supportive oncology, Multimodal intervention

## Abstract

**Introduction:**

Prehabilitation is an increasingly recognised strategy designed to optimise patients’ physical, nutritional, and psychological status before major surgery. While the benefits of trimodal prehabilitation have been documented, we developed and implemented a quadrimodal model that incorporates occupational therapy as a fourth component. This comprehensive intervention targets frail patients with advanced oncogynecologic disease, a population at high risk for postoperative complications.

**Material and methods:**

The study recruited 40 patients, 32 of whom successfully completed an intensive 3-week prehabilitation programme. The programme comprised four modules: physiotherapy, nutritional support, psychological support, and occupational therapy. Patients completed 4 days of inpatient care and 3 days of home care per week. The effectiveness of the programme was assessed using a range of methods, including physical tests (6-min walk test, 5 × sit-to-stand test), psychological status questionnaires (perceived stress scale) and nutritional assessment using established questionnaires (MUST and PONS).

**Results:**

The results demonstrated statistically significant enhancements in physical fitness assessments, a decline in the frailty index, and an augmentation in psychological well-being. Notably, three patients no longer fulfilled the frailty criteria at the conclusion of the programme. Spirometric parameters, including FVC, FEV1, and PEF, exhibited substantial improvement. The nutritional intervention resulted in a reduction in the number of patients at high risk of malnutrition. Additionally, there was a notable improvement in the nutritional parameters.

**Conclusion:**

This quadrimodal prehabilitation model, integrating occupational therapy, demonstrated effectiveness in preparing frail oncologic patients for radical surgery. The approach not only improved physical and psychological readiness but also allowed for patient stratification into responders and non-responders. Its implementation in clinical practice could enhance patient outcomes and reduce the burden of postoperative recovery.

## Introduction

Prehabilitation, defined as a preoperative intervention aimed at enhancing the physical, nutritional, and mental status of patients, has demonstrated promising results across a range of surgical specialties. Trimodal prehabilitation can be characterised as a comprehensive prehabilitation approach that integrates three key modalities to optimise a patient’s physical and mental readiness for surgery. These modalities include exercise training (physiotherapy), nutritional optimisation, and psychological support. Physiotherapy plays a critical role in the prehabilitation process, with a particular focus on enhancing patients’ physical and functional capacities prior to surgery. Nutritional support is a vital component of prehabilitation, aiming to optimise a patient’s physical state and recovery potential before surgery. The benefits of nutritional support include enhanced healing and recovery, correction of nutritional deficiencies, improved physical resilience, and reduced postoperative complications. Psychological support in prehabilitation is crucial for preparing patients mentally and emotionally for surgery. It serves several key purposes: reducing stress and anxiety, improving mental resilience, enhancing compliance, and promoting positive outcomes. Our study is unique in that, in addition to the standard trimodal prehabilitation, our patients were also under the care of an occupational therapist. This is a novel approach, especially for oncogynecological prehabilitation. For orthopaedic surgery patients, prehabilitation has been shown to have a significant positive impact on function, quality of life, muscle strength, and pain both pre- and post-operatively [1]. By focussing on improving cardiovascular fitness and preserving lean muscle mass, prehabilitation aims to enhance patients’ ability to withstand surgical stress and potentially improve postoperative outcomes [2]. Prehabilitation in oncology is a preoperative intervention designed to enhance patients’ physiological reserve and functional capacity before surgery [3, 4]. It was developed to address the increased surgical risks associated with frailty and to optimise postoperative recovery, particularly in older patients [3, 5]. In cancer patients, the benefits of prehabilitation have been demonstrated, including improved continence for prostate cancer patients, reduced hospital stays and complications for lung cancer patients, and enhanced mood and physical well-being for breast and prostate cancer patients [6]. The effectiveness of prehabilitation has been particularly evident in cardiovascular and abdominal surgeries [7]. A multimodal, multidisciplinary approach to prehabilitation was recommended, typically involving three sessions per week for 6 to 8 weeks prior to surgery [7, 8]. Occupational therapy (OT) plays a crucial role in rehabilitation across various domains. In the field of stroke rehabilitation, occupational therapists utilise meaningful activities to reduce functional deficits and promote independence [9]. For geriatric patients, therapy focuses on functional goals, family contexts, and community-based practice [10]. In the field of work rehabilitation, occupational therapists are responsible for the assessment and treatment of injured employees, with the aim of facilitating their return to work [11]. The scope of this field has expanded to include cancer rehabilitation, where multidisciplinary approaches and interventions addressing psychosocial outcomes, sexuality, and return-to-work have demonstrated effectiveness [12]. OT is grounded in the concept of occupation, which encompasses everyday activities that bring purpose and meaning to life [13]. In the field of pulmonary rehabilitation, OT aims to enhance activities of daily living (ADLs), instruct energy conservation techniques, and manage breathlessness [14]. Prehabilitation, defined as the enhancement of functional capacity prior to surgery to optimise post-operative outcomes, holds particular relevance in the context of head and neck cancer (HNC) care [15]. Prehabilitation programmes for HNC patients, addressing education, self-management, and ongoing rehabilitation needs, have been shown to effectively improve quality of life and reduce treatment sequelae [15]. OT’s unique focus on patients’ functional and social needs is critical in preventing hospital readmissions by addressing ADLs, instrumental ADLs, functional cognition, and psychosocial needs [16]. Despite the potential benefits, further research is needed to clearly define OT’s role and contributions in multidisciplinary rehabilitation teams [14]. While trimodal prehabilitation in oncogynecology has been and is the subject of many ongoing studies, publications dealing with quadrimodal prehabilitation, where the fourth element is the previously mentioned occupational therapy, are currently lacking. The present study concentrated on the most high-risk patients, i.e. fragile patients with advanced oncogynecological disease. The frailty of individual patients was assessed using a frailty index composed of frailty indicators from many other previously published papers (see Table 1).

**Table 1:** Frailty index

Type of test	Number of points
Timed up and go	< 15 s = 0 ≥ 15 s = 1
Katz’s score	0 points = 0 ≥ 1 point = 1
History of patient falls	No = 0 yes = 1
Grip strength	No = 0 yes = 1
Charlson Index	< 3 points = 0 ≥ 3 points = 1
Anemia (hematocrit)	≥ 35% = 0 < 35% = 1
Albumin	3,4 = 0 < 3,4 = 1
Mini cog test	> 3 points = 0 ≤ 3 points = 1

A total score ≥ 2 indicates a fragile patient

## Material and methods

**Fig. 1:**
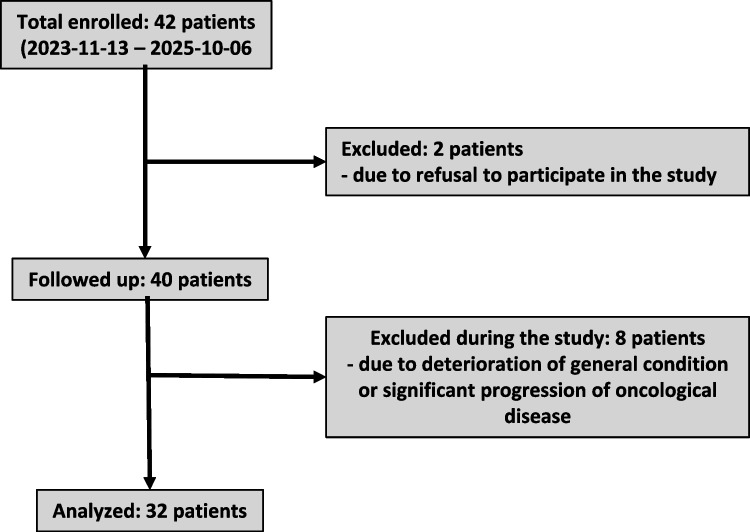
CONSORT diagram

A total of 42 patients were enrolled in the study; two patients refused to participate, and eight patients were excluded during the study (see CONSORT diagram, see Fig. 1). This prospective interventional non-randomised study recruited all patients scheduled for an extensive resection or exenteration procedure for advanced oncogynecologic cancer. Concurrently, patients were enrolled in the study who were evaluated as “frail” using a frailty index assessment (see Table 1). The patients completed an intensive quadrimodal prehabilitation programme over a 3-week period, divided into 4 days of inpatient care and 3 days of home care. The study was conducted over a 13-month period, from November 2023 to December 2024. The prehabilitation training programme comprised four modules: physiotherapy, nutritional support, psychological support, and occupational therapy.

### Description of physiotherapist interventions

Physiotherapy was delivered 4 days per week, twice daily for 20–30 min, while on the remaining 3 days, patients followed an individualised home-based exercise programme. The inpatient intervention focussed on improving physical fitness, respiratory function, and overall functional capacity prior to the planned surgical procedure. The programme included aerobic and resistance training, breathing exercises utilising inspiratory muscle training devices, and functional exercises targeting major muscle groups. Each patient received individualised education on her home exercise regimen, which included daily breathing exercises, mobilisation, and strengthening routines, as well as a prescribed daily step goal as a form of aerobic activity. Step count was monitored using a wearable activity tracker.

### Description of occupational therapist interventions

Occupational therapy interventions within the prehabilitation programme focussed on supporting functional independence and self-sufficiency through targeted training in activities of daily living (ADL) and mainly instrumental activities of daily living (IADL). A comprehensive functional assessment was performed, including the ICF classification, the hand grip test, and the WHODAS 2.0, which served as the basis for an individualised therapeutic plan. Interventions were delivered twice daily for 20–30 min and included training in personal hygiene, dressing, self-care, safe bed and out-of-bed mobility, as well as strengthening of weakened muscle groups and exercises to improve seated stability as a functional working position. Cognitive support was provided through memory training, vocabulary tasks and problem-solving activities, and psychological well-being was promoted using relaxation techniques, mindfulness, stress-reduction strategies, energy-management principles, and two weekly Feldenkrais Method sessions aimed at enhancing body awareness and neuromotor coordination. Patients received education on home-environment ergonomics and were introduced to appropriate assistive devices, such as sock aids, bath seats, and reachers, with practical training in their use. Each patient was also provided with an instructional booklet to support the continuation of therapeutic exercises in the home setting. All interventions were continuously adapted according to the patient’s functional level with the goal of optimising independence, maintaining physical and psychological readiness for surgery, and enhancing overall quality of life.

### Using ICF to assess the degree and possible improvement of disability

The International Classification of Functioning, Disability and Health (ICF) was employed as a standardised framework to quantify the degree of functional impairment and to monitor changes in patients’ functioning throughout the prehabilitation programme. Twenty clinically relevant ICF categories were selected a priori, comprising ten body functions and ten activity and participation domains (Table 2). Each category was scored using the WHO-recommended 0–4 ICF qualifier scale, which allows sensitive detection of both baseline severity and clinically meaningful changes over time and captures limitations in both capacity and performance. The selection of these 20 categories was informed by established methodology for developing clinical ICF Core Sets and reflected domains most frequently affected in women with gynaecological malignancies. These included energy, sleep, cognitive and emotional functions, pain, pelvic organ-related symptoms, musculoskeletal functions, daily activities, mobility, self-care, and psychosocial and economic roles. The chosen set therefore represents an optimal balance between comprehensiveness, clinical relevance, and feasibility in a preoperative setting, enabling standardised and reproducible assessment of functional status and its improvement within the biopsychosocial framework of the ICF.

**Table 2:** List of selected body functions (b) and activities and participation domains (d) relevant to gynecologic oncology

Code: body functions (b)	Description (b)
b130	Energy and drive functions
b134	Sleep functions
b144	Memory functions
b152	Emotional functions
b180	Experience of self and time functions
b280	Sensation of pain
b620	Urination functions
b640	Sexual functions
b710	Mobility of joint functions
b730	Muscle power functions
Code: activities and participation (d)	**Description (d)**
d230	Carrying out daily routine
d240	Handling stress and other psychological demands
d410	Changing basic body positions
d450	Walking
d520	Caring for body parts
d530	Toileting
d570	Looking after one’s health
d760	Family relationships
d770	Intimate relationships
d870	Economic self-sufficiency

Scoring system: each category is rated on a scale from 0 to 4, with the corresponding percentage ranges as follows: 0, no problem (0–4%); 1, mild problem (5–24%); 2, moderate problem (25–49%); 3, severe problem (50–95%); 4, complete problem (96–100%); Sládková P, Brtnický T, Hormandlová L, Polková M, Malina V, Koliba P, Zikán M, Hubka P, Kabele P, Dubová O, Tichá M. (2025). Occupational therapy in oncogynecology –⁠ a pilot study. Ceska Gynekol [Czech Gynecology], 90(2), 113–121. https://doi.org/10.48095/cccg2025113

### Description of psychologist interventions

Psychological care within the prehabilitation programme focussed on systematic psychodiagnostic assessment followed by targeted psychotherapeutic support. The psychologist evaluated the level of perceived stress using the PSS-10 scale in combination with a structured clinical interview, which provided a deeper understanding of how patients experience and respond to the demands of their oncologic condition. The psychotherapeutic component offered a safe and confidential setting in which patients could express anxiety, fear, and uncertainty related to the diagnosis, the upcoming surgical procedure, and disruptions to their personal and family lives. Therapeutic work centred on supporting emotional regulation, facilitating acceptance of health-related changes, and addressing issues related to identity, intimate and partner relationships, and sexuality. The psychologist employed individual psychotherapy, elements of crisis intervention, cognitive-behavioural techniques, relaxation and breathing exercises, mindfulness practices, and guided imagery, all of which contributed to reducing anxiety, improving psychological adaptation, and strengthening emotional resilience.

### Description of nutritional intervention

Nutritional assessment consisted of blood sampling and evaluation of two widely used nutritional questionnaires (MUST and PONS scores). These results were used to stratify patients according to their risk of malnutrition. Patients received nutritional interventions primarily to address anaemia, hypoproteinaemia, or vitamin deficiency, and those identified as being at high nutritional risk were additionally provided with supplemental parenteral nutrition. All patients received nutritional supplements enriched with omega-3 unsaturated fatty acids. Some studies have demonstrated immunomodulatory effects of omega-3 fatty acids, including down-regulation of inflammatory responses and improved immune function [17, 18].

All patients also completed the EORTC-QLQ-C30 International Standardised Quality of Life Questionnaire for Cancer Patients. The prehabilitation programme was subsequently integrated and implemented in our clinic into the already established ERAS (enhanced recovery after surgery) programme.

The characteristics of the patient cohort are shown in Table 3.

**Table 3:** Characteristics of the patient cohort

Characteristic	***N*** = 32^1^
**Date of admission**	
Min–max	2023–11–13–2025–10-06
**Performance status**	
0	6 (18.8%)
1	16 (50.0%)
2	6 (18.8%)
2–3	3 (9.4%)
3	1 (3.1%)
**ASA score**	
*N* non-missing	32
Mean (SD)	2.53 (0.67)
Median (Q1–Q3)	3.00 (2.00–3.00)
**BMI**	
*N* non-missing	32
Mean (SD)	30 (7)
Median (Q1–Q3)	28 (24–34)
**Age**	
*N* non-missing	32
Mean (SD)	69 (9)
Median (Q1–Q3)	70 (63–75)
**Diagnosis**	
Cervical cancer	2 (6.2%)
Endometrial cancer	9 (28.1%)
HGSC	16 (50.0%)
Borderline ovarian tumor	1 (3.1%)
Atypical endometrial hyperplasia	1 (3.1%)
Recurrent ovarian leiomyosarcoma	1 (3.1%)
Recurrent endometrial cancer	1 (3.1%)
Benign ovarian tumour	1 (3.1%)
**Hypertension**	18 (56.2%)
**Fat metabolism disorders**	8 (25.0%)
**Diabetes mellitus**	6 (18.8%)
**Asthma**	3 (9.4%)
**Date of surgery**	
Min–max	2023–12-06–2025–10–29
**Length of surgery (min)**	
*N* non-missing	32
Mean (SD)	159 (90)
Median (Q1–Q3)	123 (90–208)
**Date of discharge**	
Min–max	2023–12–14–2025–11-10
**Length of hospitalization (days)**	
*N* non-missing	31
Mean (SD)	10.4 (6.3)
Median (Q1–Q3)	9.0 (7.0–12.0)

^1^*n* (%)

### Statistical analyses

Baseline, preoperative, and postoperative values were summarised using medians due to the skewed distribution of most variables. Differences between these time points were presented as means with 95% confidence intervals, as well as medians. Statistical testing of paired differences was performed using a one-sample Wilcoxon test. For categorical data, the McNemar–Bowker symmetry test was used to assess changes in scores. The analysis was performed using R software (version 4.3.2). All tests were two-sided and conducted at a 5% significance level. No correction for multiple comparisons was applied; therefore, *p*-values should be interpreted as nominal.

## Results

Of the 40 patients who entered the study, 32 completed the entire intensive prehabilitation programme. The eight aforementioned patients did not complete the programme, as their condition did not improve despite the intensive efforts of the entire team. These patients were classified as non-responders. The primary objective of the study was to evaluate the changes in the monitored parameters across all four modalities. Moreover, an assessment was undertaken of the changes in the patients’ quality of life using the internationally validated cancer questionnaire EORTC-QLQ-C30. A comparison was made of the values obtained prior to the intensive quadrimodal prehabilitation programme (“baseline data”) with those obtained post-completion of the programme, i.e., preoperatively (“Pre-Op”). For the purpose of the psychological evaluation, the psychological state of the patients was also assessed 3 months after radical surgery (“Post-Op”).

### Frailty index

**Fig. 2:**
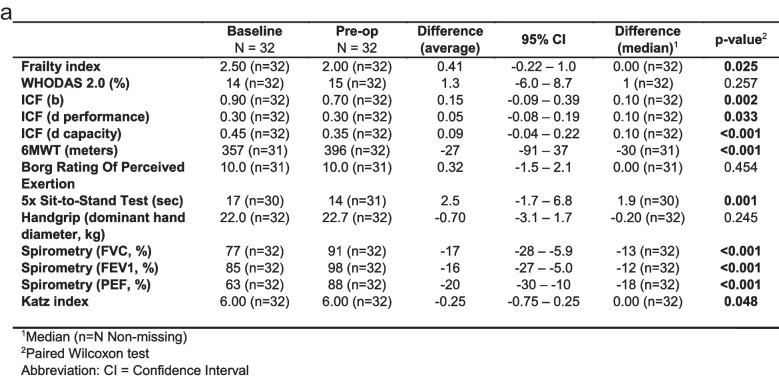

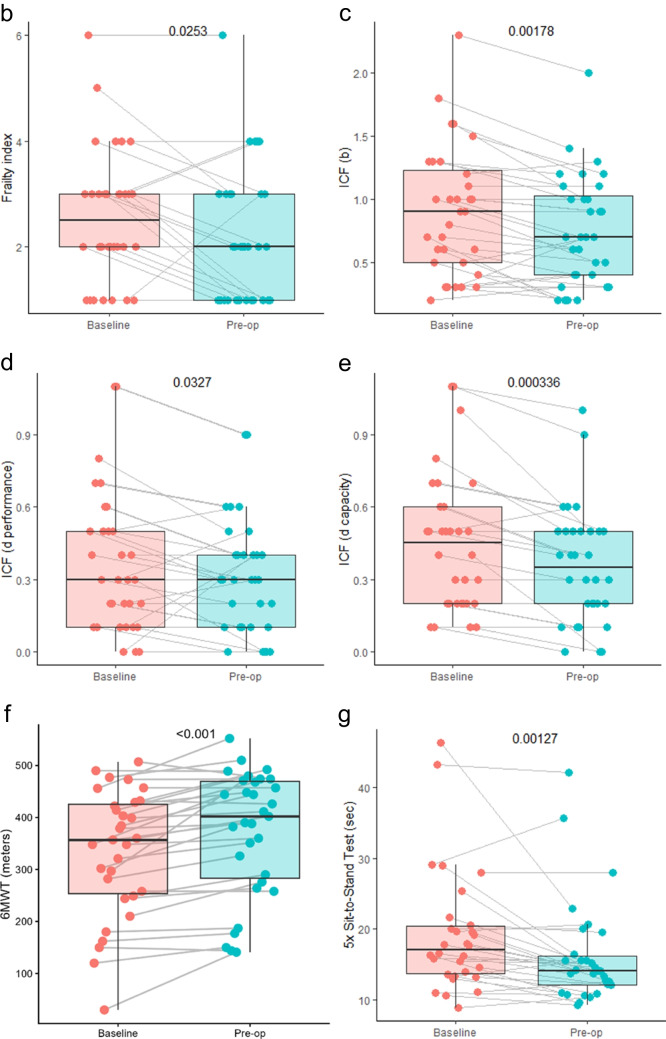

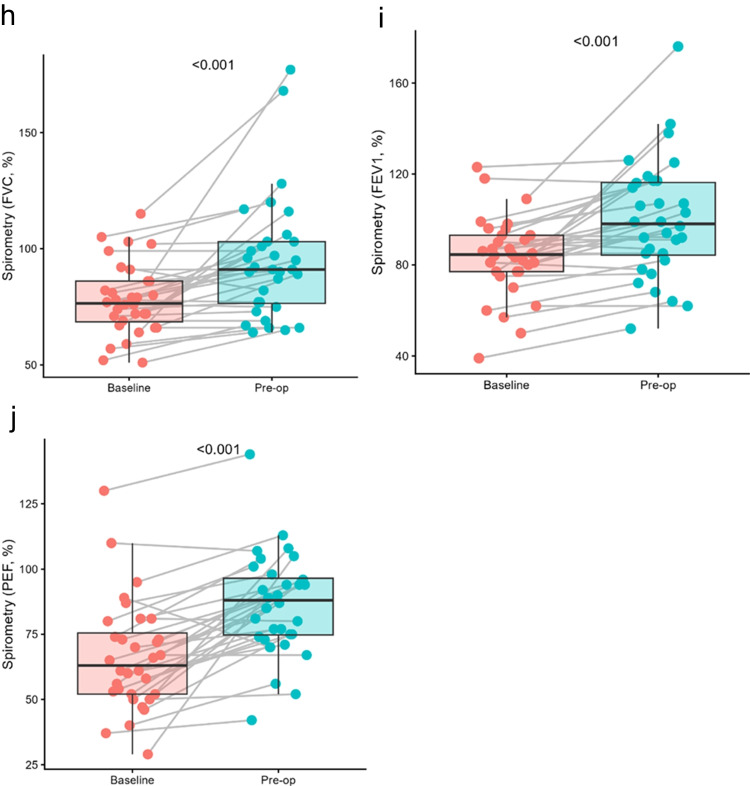
Assessed Frailty and psychological and occupational parameters: **a** overview of the tests, **b** frailty index, **c** ICF (b), **d** ICF (d performance), **e** ICF (d capacity), **f** 6MWT (meters), **g** 5 × sit-to-stand test (sec), **h** spirometry (FVC, %), **i** spirometry (FEV1, %), and **j** spirometry (PEF, %)

At the beginning of the prehabilitation programme, 25 out of 32 patients were assessed as “frail”, i.e., Frailty Index score ≥ 2 (see Fig. 1). At the conclusion of the prehabilitation programme, the Frailty Index was positive in only 19 patients. This indicates that as a result of the prehabilitation intervention, 6 patients demonstrated such improvement that they no longer fell into the frailty group. The improvement in the Frailty Index was found to be statistically significant (see Fig. 2a and b).

### Evaluated parameters of physiotherapy and occupational therapy

The findings of the performance tests demonstrated a marked enhancement on the 6-min walk test (6MWT), indicating a statistically significant increase in the distance walked by the patient within the stipulated time frame. The 5 × sit-to-stand test demonstrated a marked enhancement in patient fitness, accompanied by a statistically significant reduction in the time required to complete the exercise. However, the most substantial changes were observed in spirometric values, where a significant statistical improvement was evident in all three parameters studied. An improvement in the International Classification of Functioning, Disability and Health (ICF) scores was observed in all three occupational therapy parameters. Conversely, no improvement was evident in the Borg Rating of Perceived Exertion, Katz index and WHODAS 2.0 questionnaires (see Fig. 2a and c–j).

### Assessed psychological parameters

**Fig. 3:**
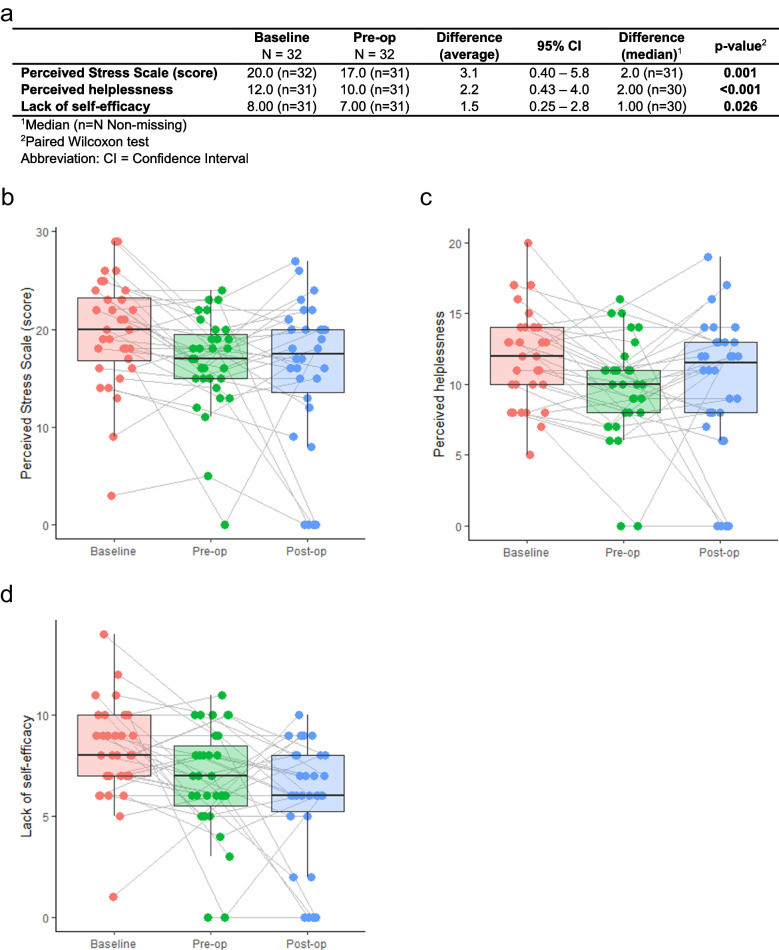
Assessed psychological parameters: **a** overview of the tests, **b** perceived stress scale (score), **c** perceived helplessness, and **d** lack of self-efficacy

The results of the study demonstrated a statistically significant enhancement in the psychological well-being of the patients, as indicated by the Perceived Stress Scale (PSS-10). This observation was evident in both the overall assessment of the questionnaire and its specific subscales, including perceived helplessness and lack of self-efficacy (see Fig. 3a–d).

### Assessed the nutritional parameters

**Fig. 4:**
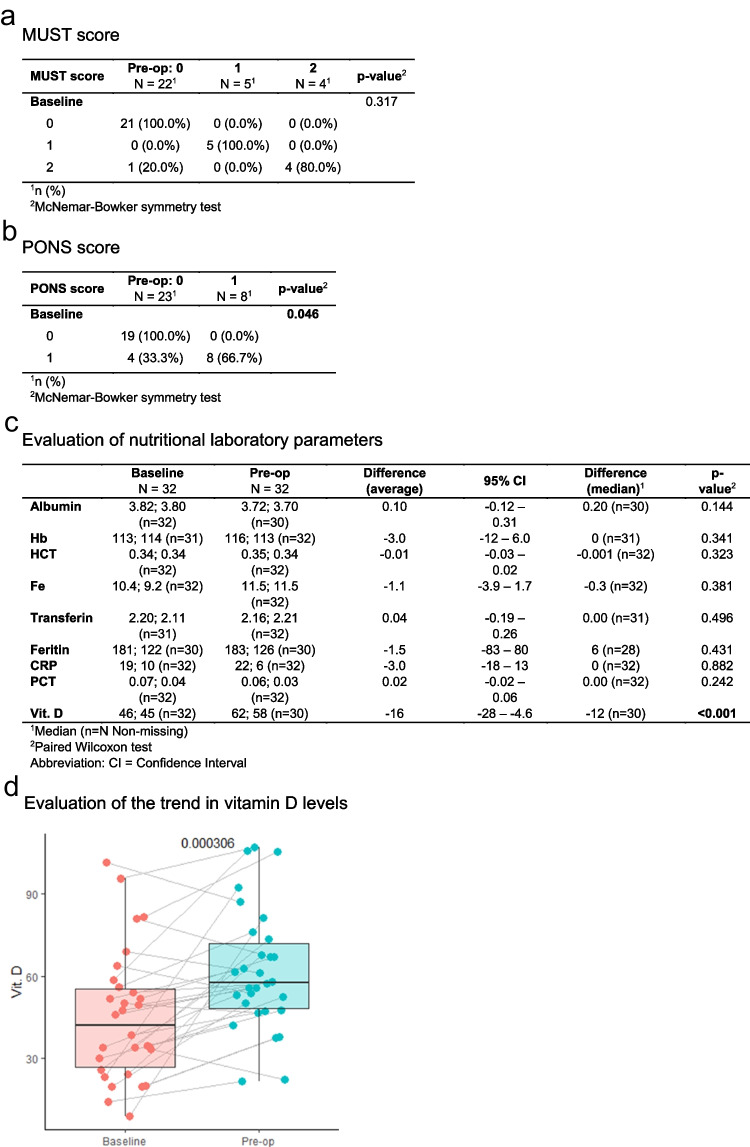
Assessed nutritional parameters: **a** MUST score, **b** PONS score, **c** evaluation of nutritional laboratory parameters, and **d** evaluation of the trend in vitamin D levels

Following analysis of the input parameters from the MUST nutritional questionnaire, it was determined that five patients were at moderate risk of malnutrition, while five patients were at high risk. Upon completion of the prehabilitation programme, the risk levels remained unchanged for the five patients at moderate risk, and the number at high risk decreased to four patients. As reflected in the PONS questionnaire, there was a significant enhancement in the nutritional status of patients. At the initial stage, twelve patients were identified as being nutritionally at risk, while this number decreased to eight at the preoperative assessment stage (see Fig. 4a and b). Of the nutritional blood tests studied, only two patients had an albumin level below 3.0 dg/l at baseline, before the nutritional intervention (the cut-off of the PONS questionnaire). However, after the nutritional intervention, all patients had a blood albumin level above 3.0 dg/l. A total of fifteen patients had mild hypovitaminosis D (20–50 nmol/L) and three patients had severe hypovitaminosis D (below 20 nmol/L) at baseline. Following the implementation of a nutritional intervention, only seven patients demonstrated mild hypovitaminosis D on preoperative blood tests, and none exhibited severe hypovitaminosis D. The improvement in vitamin D values after the nutritional intervention was found to be statistically significant (see Fig. 4c and d).

### EORTC-QLQ-C30 quality of life questionnaire assessment

Assessment using the internationally validated EORTC QLQ-C30 questionnaire demonstrated overall stability in health-related quality of life between baseline and the completion of prehabilitation. No statistically significant changes were observed in most functional and symptom domains, including the global health status/QoL score. A statistically significant reduction in pain was observed after prehabilitation, indicating an improvement in this symptom, while all other domains remained unchanged (see Table 4).

**Table 4:** Quality of life assessment (EORTC-QLQ-C30)

	Baseline *N* = 32	Pre–op *N* = 32	Difference (average)	95% CI	Difference (median)^1^	*p*-value^2^
QL	59.9 (23.6) 50 (42–83) (*n* = 26)	65.3 (19.3) 67 (50–83) (*n* = 24)	− 5.3	− 18 to 6.9	0 (*n* = 22)	0.533
PF	73.2 (18.7) 80 (67–87) (*n* = 29)	74.7 (21.9) 80 (67–87) (*n* = 25)	− 1.4	− 13 to 9.8	0 (*n* = 24)	0.761
RF	76.4 (25.4) 83 (67–100) (*n* = 29)	67.4 (29.3) 67 (50–100) (*n* = 24)	9.1	− 6.2 to 24	0(*n* = 24)	0.324
EF	73.7 (24.3) 79 (58–92) (*n* = 26)	78.9 (20.3) 83 (67–100) (*n* = 25)	− 5.2	− 18 to 7.4	0 (*n* = 23)	0.243
CF	84.0 (19.1) 83 (83–100) (*n* = 26)	86.7 (17.3) 83 (83–100) (*n* = 25)	− 2.7	− 13 to 7.6	0.0 (*n* = 23)	0.121
SF	74.7 (30.1) 83 (67–100) (*n* = 25)	73.3 (27.2) 83 (50–100) (*n* = 25)	1.3	− 15 to 18	0 (*n* = 22)	0.842
FA	33.9 (20.4) 33 (22–33) (*n* = 29)	29.3 (23.8) 33 (11–33) (*n* = 25)	4.6	− 7.6 to 17	0 (*n* = 24)	0.354
NV	5.4 (12.9) 0 (0–0) (*n* = 28)	2.0 (7.3) 0 (0–0) (*n* = 25)	3.4	− 2.4 to 9.1	0 (*n* = 23)	0.371
PA	24.1 (24.6) 17 (0–33) (*n* = 29)	14.0 (21.9) 0 (0–17) (*n* = 25)	10	− 2.6 to 23	0 (*n* = 24)	0.015
DY	18.4 (24.5) 0 (0–33) (*n* = 29)	14.7 (23.7) 0 (0–33) (*n* = 25)	3.7	− 9.5 to 17	0 (*n* = 24)	0.407
SL	37.9 (31.8) 33 (0–67) (*n* = 29)	26.7 (27.2) 33 (0–33) (*n* = 25)	11	− 4.8 to 27	0 (*n* = 24)	0.112
AP	14.9 (22.9) 0 (0–33) (*n* = 29)	12.5 (19.2) 0 (0–33) (*n* = 24)	2.4	− 9.2 to 14	0 (*n* = 23)	> 0.999
CO	14.3 (19.1) 0 (0–33) (*n* = 28)	13.3 (23.6) 0 (0–33) (*n* = 25)	0.95	− 11 to 13	0 (*n* = 23)	> 0.999
DI	2.6 (9.1) 0 (0–0) (*n* = 26)	5.3 (12.5) 0 (0–0) (*n* = 25)	− 2.8	− 8.9 to 3.4	0(*n* = 23)	0.149
FI	14.7 (25.6) 0 (0–33) (*n* = 25)	14.7 (29.0) 0 (0–0) (*n* = 25)	0.00	− 16 to 16	0 (*n* = 23)	> 0.999
QLQTOTAL	80.5 (11.7) 84 (72–90) (*n* = 25)	83.0 (10.8) 82 (73–93) (*n* = 23)	− 2.5	− 9.0 to 4.1	− 3 (*n* = 22)	0.205

*CI* confidence interval, *QL* quality of Life, *PF* physical functioning, *RF* role functioning, *EF* emotional functioning, *CF* cognitive functioning, *SF* social functioning, *FA* fatigue, *NV* nausea and vomiting, *PA* pain, *DY* dyspnea, *SL* insomnia, *AP* appetite loss, *CO* constipation, *DI* diarrhea, *FI* financial difficulties

^1^Median (*n* = *N* non-missing)

^2^Paired Wilcoxon test

## Discussion

One of the aims of the study was to identify patients at the highest risk of postoperative complications. These patients were characterised by advanced age, a risk of malnutrition, and the undertaking of radical surgery for cancer. Frailty is defined as a multidimensional syndrome characterised by decreased physiological reserve and increased vulnerability to stressors [19]. This concept is particularly relevant in the context of older surgical patients. As demonstrated by Makary et al. [20], there is an increased risk of postoperative complications, prolonged hospital stays, and higher mortality rates. Furthermore, the frailty assessment enhances preoperative risk evaluation and can predict outcomes better than traditional risk models [20, 21]. A variety of tools exist for the assessment of frailty, including the Clinical Frailty Scale and the Frailty Index [21]. The identification of frail patients prior to surgery allows for targeted interventions, such as prehabilitation, nutritional support, and the prevention of delirium [21]. As the ageing population increases, understanding and addressing frailty becomes crucial for improving surgical outcomes and patient care [22, 23]. Consequently, it appears highly probable that this cohort of patients at risk will greatly benefit from the prehabilitation programme [24].

There was a significant improvement in patients’ physical condition, as demonstrated by improvements in 6MWT and 5 × sit-to-stand test parameters. This improvement was not only statistically significant but also positively perceived by the patients themselves. Another important result was the significant improvement in all three spirometric parameters (FVC, FEV1, PEF). A rapid recovery in respiratory function post-surgery is a favourable prognostic factor for a faster transfer of patients from the ICU to a standard room, as well as reducing the incidence of postoperative complications [25].

A 3-week, inpatient, intensive quadrimodal prehabilitation programme for 32 women with malignant gynaecological cancers resulted in a small but statistically significant reduction in the severity of problems across key ICF domains of body functions and activities/participation (both performance and capacity) [26]. These findings are consistent with the ICF framework and with current literature on multimodal prehabilitation in oncology. The selection of 20 ICF categories reflects both the recommended methodological procedures for developing ICF Core Sets and the most common clinical priorities in this patient population. Based on these results, the International Classification of Functioning, Disability and Health (ICF) can be recommended as an appropriate tool for determining the degree of disability and for objectively monitoring changes in functional abilities. It proved essential that an occupational therapist—given their expertise in the analysis of activities of daily living—played a central role in designing the clinical ICF form tailored for women with oncogynecologic malignancies [27].

In contrast, assessment with the EORTC QLQ-C30 questionnaire demonstrated overall stability of health-related quality of life when comparing baseline and post-prehabilitation results. Pain was the only domain showing a statistically significant improvement, while no significant changes were observed in the remaining functional and symptom scales. Given that the EORTC QLQ-C30 is primarily designed to capture the impact of cancer and oncological treatment on quality of life, it may be less sensitive to short-term functional changes associated with a relatively brief, 3-week prehabilitation intervention.

The most significant difference between this programme and other prehabilitation studies is the intensive involvement of occupational therapy. The findings of the statistical analyses indicate that this could be a promising pathway for the future development of prehabilitation programmes. In the final evaluation, the benefit of occupational therapy is evident in its role as a complementary intervention that bridges the gap between the care of the physiotherapist and the psychologist. Another important factor that makes not only the prehabilitation programme but also the ERAS programme of interest to medical staff and hospital management is that consistent pre-operative preparation significantly reduces the incidence of post-operative complications, shortens the length of hospital stay, and leads to a reduction in the number of acute unplanned follow-ups of patients in home care after discharge [28, 29]. It is thus evident that the economic dimension of the project represents a notable factor. Patient preference for holistic, whole-person care rather than narrowly specialised treatment aligns closely with the philosophy of multimodal prehabilitation and may help explain the low drop-out rate observed in the study*.* None of the patients requested to be withdrawn from the study during its course. However, six patients discontinued their participation in the study due to either a deterioration in their general internal condition or cancer progression. In all cases, the decision was made by consensus of the prehabilitation team, not as a result of the patient’s wish to end the programme.

In our view, the design of the programme was a key contributing factor to the high level of adherence exhibited by patients. Having patients spend 4 days in hospital and 3 days at home proved to be an effective approach. This allowed for a more intensive programme during their hospitalisation, while also enabling patients to remain with their loved ones and in their familiar home environment. Conversely, data concerning prehabilitation programmes that primarily offer home-based or tele-rehabilitation services demonstrate significantly lower adherence rates, with dropout rates ranging from 37 to 84% of patients [30].

This study has several strengths and limitations. The main strengths include the novel expansion of the conventional trimodal prehabilitation model through the incorporation of occupational therapy, along with a comprehensive assessment of physical, nutritional, and psychological domains. The intervention was associated with improvements in key clinically relevant parameters, including functional capacity and frailty status. In addition, the structured yet pragmatic design of the programme, combining supervised inpatient interventions with individualised home-based training, supports its feasibility and applicability in routine clinical practice. Several limitations should be acknowledged. The study cohort was limited in size, which may restrict the generalizability of the findings. Furthermore, the single-centre design, absence of randomisation and a control group, and the lack of long-term postoperative follow-up limit causal inference and the ability to assess sustained postoperative outcomes. Despite these limitations, the results indicate a reproducible trend across key outcomes, warranting further investigation of this quadrimodal prehabilitation approach in future controlled studies of frail oncogynecologic patients.

## Conclusion

This study demonstrates the feasibility of implementing a quadrimodal prehabilitation programme integrating physiotherapy, nutritional support, psychological counselling, and occupational therapy in frail oncologic patients undergoing complex surgical treatment. The inclusion of occupational therapy, which is not routinely incorporated into prehabilitation pathways, was intended to strengthen functional independence and activities of daily living as part of preoperative optimization. Within this context, occupational therapy targeted essential functional areas such as energy management, cognitive functioning, emotional regulation, and the performance of ADL and mainly IADL through individualised training and structured therapeutic tasks. By helping patients organise daily activities, manage fatigue and anxiety, and maintain basic self-care and mobility, these interventions likely contributed to the functional gains observed, particularly among frail patients who tend to have limited physiological and functional reserve before surgery. Because both therapeutic planning and outcome evaluation were aligned with the same set of twenty clinically relevant ICF categories, even modest improvements in capacity and performance were consistently captured.

The intervention was associated with improvements in several clinically relevant domains, including physical performance, frailty status, psychological well-being, and nutritional parameters. Beyond overall patient optimization, the programme enabled the identification of differential responses to prehabilitation, suggesting potential value in distinguishing between patients who benefit from short-term intensive preoperative optimization and those who demonstrate limited functional response. In this context, prehabilitation may function as a form of preoperative functional stress test, informing multidisciplinary surgical decision-making and, in selected cases, supporting consideration of alternative or modified treatment strategies rather than proceeding with radical surgery. These findings indicate that prehabilitation may represent a supportive component in preoperative assessment rather than a stand-alone determinant. While causal conclusions and long-term clinical outcomes cannot be inferred, the results support further integration and evaluation of comprehensive prehabilitation strategies, including occupational therapy, within perioperative care frameworks such as Enhanced Recovery After Surgery (ERAS). By enhancing patients’ functional and psychological reserve prior to surgery, quadrimodal prehabilitation may contribute to improved perioperative resilience, warranting validation in future adequately powered, controlled studies.

## Data Availability

No datasets were generated or analysed during the current study.

## Abbreviations

OT: Occupational therapy
ADLs: Activities of daily living
HNC: Head and neck cancer
6MWT: Six-min walk test
ICF: International Classification of Functioning, Disability and Health
WHODAS: World Health Organization Disability Assessment Schedule
PSS-10: Perceived Stress Scale
EORTC QLQ-C30: European Organisation for Research and Treatment of Cancer Quality of Life Questionnaire-Core 30
ERAS: Enhanced recovery after surgery
FVC: Forced vital capacity
FEV1: Forced expiratory volume in 1 s
PEF: Peak expiratory flow
